# Global Mortality Burden of Cirrhosis and Liver Cancer Attributable to Injection Drug Use, 1990–2016: An Age-Period-Cohort and Spatial Autocorrelation Analysis

**DOI:** 10.3390/ijerph15010170

**Published:** 2018-01-22

**Authors:** Jin Yang, Yunquan Zhang, Lisha Luo, Runtang Meng, Chuanhua Yu

**Affiliations:** 1 Department of Epidemiology and Biostatistics, School of Health Sciences, Wuhan University, 185 Donghu Road, Wuhan 430071, China; jinan0218@163.com (J.Y.); Yun-quanZhang@whu.edu.cn (Y.Z.); 13006362970@163.com (L.L.); mengruntang@163.com (R.M.); 2 Global Health Institute, Wuhan University, 8 Donghunan Road, Wuhan 430072, China

**Keywords:** injection drug use, cirrhosis, liver cancer, mortality burden, age-period-cohort model analysis, spatial autocorrelation analysis

## Abstract

We analyzed the temporal and spatial variations in mortality burden of cirrhosis and liver cancer attributable to injection drug use (IDU) from 1990 to 2016. Mortality data of IDU-attributable cirrhosis and IDU-attributable liver cancer on the global and national scales from 1990 to 2016 were collected from the Global Burden of Disease (GBD) studies. Age-period-cohort (APC) model analysis was used to analyze the global mortality trends of target disease, and spatial autocorrelation analysis based on Geographic Information System was applied to illustrate the clusters of the most epidemic countries. Globally, from 1990 to 2015, mortality rates (age-standardized, per 100,000) of IDU-attributable cirrhosis increased continually from 1.5 to 1.9, while from 0.4 to 0.9 for IDU-attributable liver cancer. The APC model analysis indicated that the increases of mortality were mainly driven by period effects, with the mortality risk increasing by 6.82-fold for IDU-attributable cirrhosis and 3.08-fold for IDU-attributable liver cancer. The spatial analysis suggested that IDU-attributable cirrhosis mortality were geographically clustered from 1990 to 2016, and hot spots were mainly located in less well developed countries of Latin America, East and Central Europe and Central Asia. Our study provides epidemiological evidence for global interventions against advanced liver disease among injection drug users (IDUs).

## 1. Introduction

Injection drug use (IDU) is not only a serious social problem, but also a public health problem that is an effective transmission route of blood-borne pathogens including HIV, hepatitis C virus (HCV) and hepatitis B virus (HBV) [1,2]. Traditional HBV/HCV transmission routes such as iatrogenic injections, blood transfusion and mother-to-child transmission have been attenuated through effective public health interventions including screening of blood donors and mass vaccination of HBV [3,4]. While these trends are encouraging, however, HCV transmission is increasingly driven by IDU, especially in developed countries [5,6,7,8,9]. The United Nations Office on Drugs and Crime (UNODC) estimated that 12 million people inject drugs in 2014, of whom, one in half are hepatitis C infected [10]. Another systematic review estimated that globally, 8% of injection drug users (IDUs) are HBsAg positive [11].

What’s worse, both HBV and HCV are able to induce a chronic infection that may lead to progressive liver fibrosis, cirrhosis, and liver cancer [12,13]. Therefore, IDU as a transmission way of HBV/HCV poses a risk for cirrhosis and liver cancer. In 2015, it is estimated that IDU as a risk factor causes 224,000 deaths via HCV infection and 7000 deaths via HBV infection, among which, cirrhosis and liver cancer resulting from chronic hepatitis infection accounts for most majority of the deaths (99.6%) [14].

Undoubtedly, IDU as an increasing predominate transmission way of HBV/HCV has become an important risk source of advanced liver disease. However, epidemiological descriptions of IDU-related liver disease in the last three decades have not included the trends and geographical distribution patterns in the mortality burden of cirrhosis and liver cancer. Since the availability of effective vaccines for HBV and the improvements in antiviral therapies for HBV and HCV make big room for the control and prevention of the advanced liver disease [15,16,17], therefore, a better understand of the trends and distribution of IDU-attributable liver disease is needed to inform global intervention strategies.

The Global Burden of Disease (GBD) 2015 and 2016 studies provide the global and country level risk-specific mortality data of cirrhosis and liver cancer [18,19]. Our study applied age-period-cohort (APC) model analysis and spatial autocorrelation analysis to IDU-specific mortality data, so as to create a complete and in-depth report detailing the trends and geographical distribution patterns of IDU-attributable cirrhosis and liver cancer mortality over the period of 1990–2016.

## 2. Materials and Methods

### 2.1. Data Sources

The Institute for Health Metrics and Evaluation (IHME) is an independent global health research center at the University of Washington, which provides rigorous and comparable measurement of the world’s most important health problems. The GBD 2015 study [18], a large international cooperation project coordinated by the IHME, provided comprehensive estimation of risk-specific mortality for 79 risk sources of death (including behavioral, environmental and occupational, and metabolic risks) on the global, regional and national scales from 1990 to 2015. The GBD 2016 study [19], based on the GBD 2015 study, provided estimation of risk-specific mortality for 84 risk sources of death from 1990 to 2016.

Age-standardized mortality rates on the global level between 1990 and 2015 were collected from the GBD 2015 study to plot the mortality trend of IDU-attributable cirrhosis and IDU-attributable liver cancer. Age-specific (15–19, …, 75–79) mortality rates of on the global level between 1990 and 2015 were collected from the GBD 2015 study for APC model analysis. Age-standardized mortality rates on national scales (195 countries) between 1990 and 2016 were collected from the GBD 2016 study for spatial autocorrelation analysis.

### 2.2. Age-Period-Cohort Analysis

Temporal trends of mortality are actuated by the merged effects of three temporal parameters: age, time period and birth cohort [20]. The age-period-cohort (APC) model as a parametric statistical model widely used in epidemiology research, estimates the independent effect of age, time period and birth cohort on disease morbidity or mortality, and provides important clues for social, historical, and environmental factors that impacts disease morbidity or mortality [21,22,23,24].

Υ = μ + α_i_ × age + β_j_ × period + γ_k_ × cohort + ε (1)

where γ denotes expectancy mortality of the i (i = 15–19, …, 75–79) age group during the j (j = 1990, 1995, 2000, 2005, 2010 and 2015) period; α_i_ represents age effect of the i age group; β_j_ represents period effect of the j period; γ_k_ represents cohort effect of the k (k = i + j − 1) birth cohort; μ is the intercept, and ε is the residual. Because there is a linear relationship between the three temporal parameters (cohort = period − age), the APC model suffered from the identifiability problem [22,23,25,26]. We used the intrinsic estimator (IE) [27,28] method, a new and promising method to deal with the identifiability problem, so as to obtain identifiable estimations for the age, period and cohort effects. To compare the relative mortality risk across ages, periods, and birth cohorts, the coefficients of age, period and cohort effects were convert into relative risk (RR):

RR_i_ = Exp (coef_i_)/Exp (coef_0_) (2)

where coef_i_ denotes coefficient of the i age, period or cohort group, coef_0_ denotes coefficient of the first age, period or cohort group, RR_i_ represents relative mortality risk of the i age, period or cohort group relative to the first age, period or cohort group. The goodness-of-fit of the APC model was evaluated by Residual Deviance, Akaike information criterion (AIC), and Bayesian information criterion (BIC). APC analyses were implemented with STATA 13.1 (StataCorp, College Station, TX, USA).

### 2.3. Spatial Autocorrelation Analysis

Neighbor geographic units tended to present similar disease epidemic pattern since neighbors share similar geographic and sociocultural environment [29]. Spatial autocorrelation analysis is often used in epidemiology research to unpack the spatial distribution patterns of disease mortality and to identify disease risk sites [30].

Firstly, data of country-level mortality rates of IDU-attributable cirrhosis and IDU-attributable liver cancer were matched to the geospatial databases of Geographic Information System (GIS) to form the spatial database. Secondly, the Global Moran’s I statistic [31] was applied to explore the overall spatial patterns of disease mortality, with Moran’s I values representing the degree of clustering or dispersing of mortality among countries. Moran’s I values range from −1 to +1, with −1 indicating perfect dispersed, +1 indicating perfect clustered and 0 indicating a random spatial pattern. For statistical hypothesis testing, Moran’s I values can be transformed to *Z*-scores, with a *Z*-score value ≥1.96 or ≤−1.96 indicating spatial autocorrelation being significant at the 95% level. Thirdly, the Getis-Ord Gi* local statistic (also known as Hot Spot Analysis) [32] was adopted to recognize the location of the clusters. Under the null hypothesis of no spatial autocorrelation, the *Z*-score, the output of the Gi* function, was assigned to each country to recognize the statistical significance of clusters. If *Z*-score value ≥1.65, ≥1.96 or ≥2.58, the local clusters were identified as high mortality—high mortality clusters (hot spots) with a significance level of 0.90, 0.95 and 0.99, respectively. If *Z*-score value ≤−1.65, ≤−1.96, or ≤−2.58, the local clusters were identified as low mortality—low mortality clusters (cold spots) with a significance level of 0.90, 0.95 and 0.99, respectively. In our study, the significance level of 0.95 was considered as statistically significant. ArcGIS (version 10.2.2, ESRI Inc., Redlands, CA, USA) was used for spatial autocorrelation analysis, with 999 permutations set for randomization.

## 3. Results

### 3.1. Global Mortality Trends of Cirrhosis and Liver Cancer Attributable to Injection Drug Use

**Figure 1:**
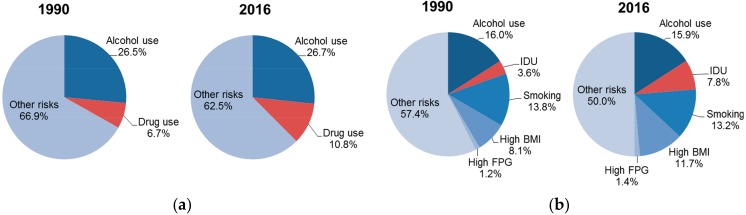
Risk-specific disease mortality on the global level in 1990 and 2016 for (**a**) cirrhosis; (**b**) liver cancer. IDU: injection drug use; BMI: body-mass index; FPG: fasting plasma glucose.

From 1990 to 2016, IDU became an increasing larger risk source of cirrhosis and liver cancer (Figure 1). Specially, IDU as a risk factor accounted for 3.6% (16,500) of the global liver cancer deaths in 1990, and this proportion increased to 7.8% (65,000) in 2016. During the same period, the proportion of cirrhosis deaths attributable to IDU increased from 6.7% (59,400) to 10.8% (141,100). Most of the deaths attributable to IDU were due to chronic hepatitis C infection (98%), while HBV infection accounted for very minor part.

**Figure 2:**
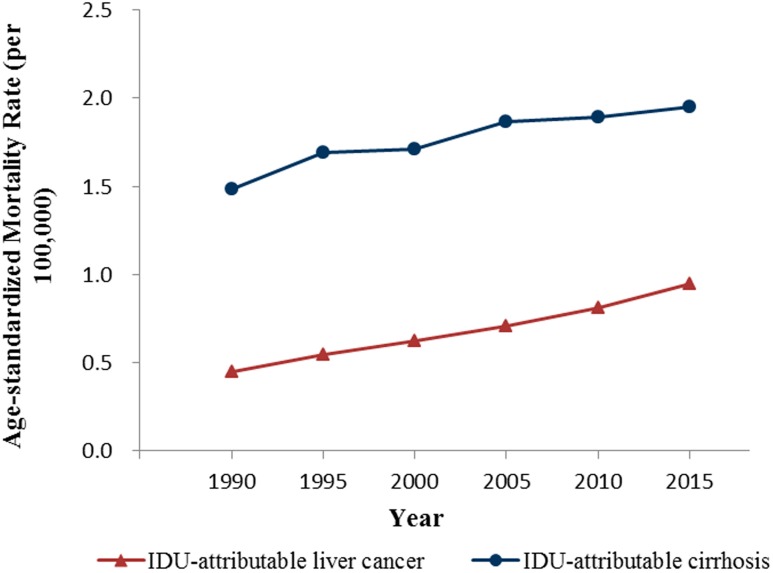
Mortality rates (age-standardized, per 100,000) of IDU-attributable cirrhosis and IDU-attributable liver cancer, 1990–2015, globally.

Mortality rates (age-standardized, per 100,000) of IDU-attributable cirrhosis and IDU-attributable liver cancer on the global level from 1990 to 2015 were shown in Figure 2. The global mortality trends were almost identical for both diseases throughout the study period, with greater mortality observed in IDU-attributable cirrhosis. Specially, from 1990 to 2015, mortality rate of IDU-attributable liver cancer increased continually from 0.4 to 0.9, while from 1.5 to 1.9 for mortality rate of IDU-attributable cirrhosis.

### 3.2. The Results of Age-Period-Cohort Model Analysis

**Figure 3:**
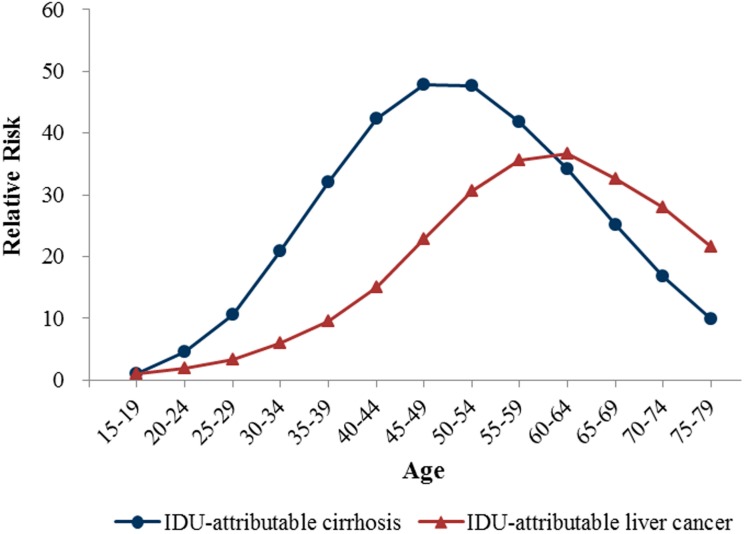
Age effects on mortality rates of IDU-attributable cirrhosis and IDU-attributable liver cancer.

**Figure 4:**
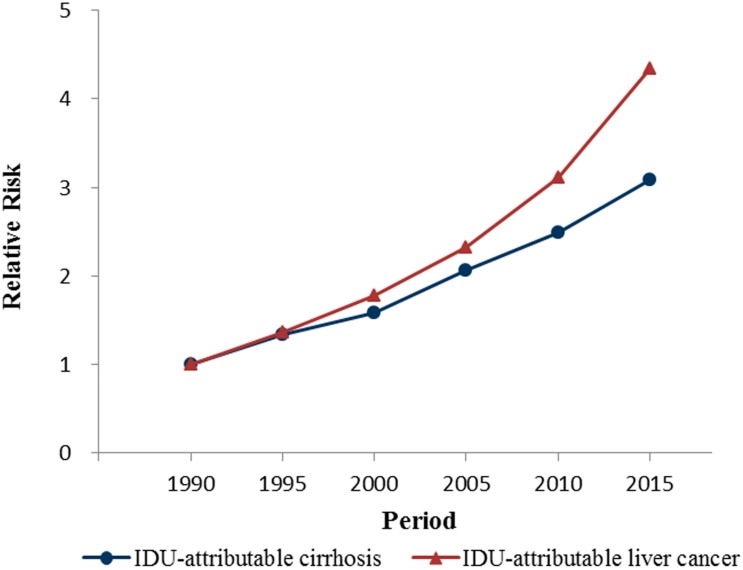
Period effects on mortality rates of IDU-attributable cirrhosis and IDU-attributable liver cancer.

**Figure 5:**
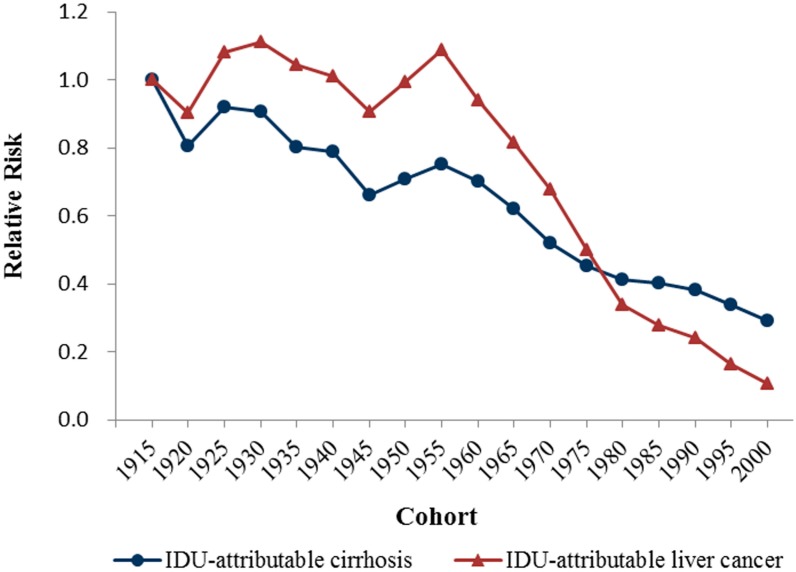
Cohort effects on mortality rates of IDU-attributable cirrhosis and IDU-attributable liver cancer.

Figure 3, Figure 4 and Figure 5 show the age, period and cohort effects of the mortality of IDU-attributable cirrhosis and IDU-attributable liver cancer based on the mortality relative risk (RR) values. The age effects for both diseases mortality were quite notable and displayed a reversed V-formed curve. For IDU-attributable cirrhosis, age coefficients peaked at 0.98 (95% CI: 0.97–0.99) at 45–49 years, and the corresponding mortality risk was 47.78 (95% CI: 45.33–50.36) times relative to the first age group (15–19 years). Compared to age effect of IDU-attributable cirrhosis, age effect of IDU-attributable liver cancer peaked at a later age (60–64 years), with the age coefficients of 1.12 (1.11–1.14) and the corresponding mortality risk of 36.63 (95% CI: 32.48–41.30).

The period effects for both diseases increased consistently and markedly during the whole period, while the increase of period effects for IDU-attributable liver cancer mortality was more rapid. From 1990 to 2015, the period effect of IDU-attributable cirrhosis increased from −1.21 to 0.54, with the mortality risk increasing by exp(0.54)/exp(−1.21) = 6.82-fold. For IDU-attributable liver cancer, the period effect increased from −0.72 to 0.79, with mortality risk increasing by exp(0.79)/exp(−0.72) = 3.08-fold.

The overall cohort effects for both diseases declined during the 1915 to 2000 birth cohorts. Specifically, the cohort effect of IDU-attributable cirrhosis declined irregularly from the 1915 birth cohort to the 1960 birth cohort, with two tiny increases in the 1910–1929 and 1945–1959 birth cohorts. Contemporaneity, the cohort effect of IDU-attributable liver cancer increased irregularly, with increases observed in the 1910–1934 and 1945–1959 birth cohorts. Cohort effects for both diseases declined markedly thereafter the 1960 birth cohort.

Overall, from the 1915 birth cohort to the 2000 birth cohort, the cohort effect of IDU-attributable liver cancer declined from 0.24 to −1.82, with the mortality risk declining by exp(0.51)/exp(−0.71) = 0.29-fold. Similarly, the cohort effect of IDU-attributable liver cancer declined from 0.24 to −1.82, with the mortality risk declining by exp(0.50)/exp(−1.73) = 0.11-fold. This suggested that, compared to people born in 1915, people born in 2000 suffered 0.55 times of mortality risk from IDU-attributable cirrhosis and 0.13 times of mortality risk from IDU-attributable liver cancer. Detailed coefficients of age, period and cohort effects and corresponding RR values were given in Supplementary Materials Tables S1 and S2.

### 3.3. Country-Level Mortality of IDU-Attributable Cirrhosis and IDU-Attributable Liver Cancer from 1990 to 2016

**Figure 6:**
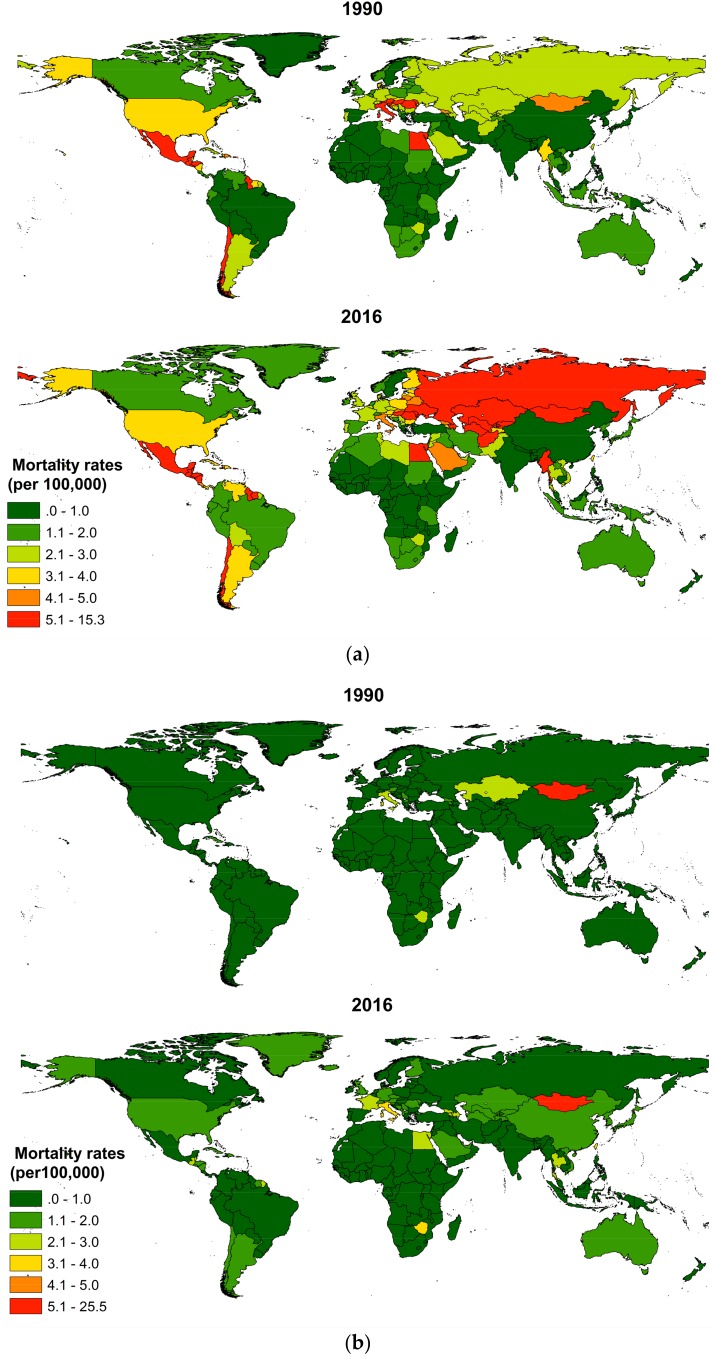
Mortality rates (age-standardized, per 100,000) of (**a**) IDU-attributable cirrhosis and (**b**) IDU-attributable cirrhosis in 1990 and 2016, by country.

Country-level mortality rates (age-standardized, per 100,000) of IDU-attributable cirrhosis and IDU-attributable liver cancer in 1990 and 2016 were shown in Figure 6. For IDU-attributable cirrhosis mortality (Figure 6a), Guyana and Mexico in South Latin America, Egypt in North Africa, and Myanmar in South East Asia were countries with a traditionally high mortality since 1990. From 1990 to 2016, great increase of mortality was observed in Europe countries of Russia, Ukraine Romania and Hungary, and Asia countries of Mongolia, Kazakhstan, Turkmenistan, Uzbekistan, and Afghanistan, while mortality kept in a low level in most of the African countries. In 2016, high mortality countries were Guyana (9.9) and Mexico (8.7) in South Latin America; Moldova (15.3), Romania (7.4), Hungary (6.5) Ukraine (6.3) and Russia (5.8) in Europe; Mongolia (8.8), Kazakhstan (8.3), Turkmenistan (7.6), Uzbekistan (6.8), Afghanistan (5.3) and Myanmar (6.9) in Asia; and Egypt (9.5) in Africa. However, the highest deaths were in the United States (17,215), China (13,265) and Russia (11,187), which altogether accounted for 29.5% of the global IDU-attributable cirrhosis deaths.

Contrast with cirrhosis, IDU made a smaller contribution to mortality burden of liver cancer in most of the countries (Figure 6b). In 2016, the highest mortality rate for IDU-attributable liver cancer was observed in Mongolia (25.3), which far surpasses other countries. Mortality rate following Mongolia, among others, were Zimbabwe (3.7), Italy (3.4), Egypt (2.8), France (2.2), Thailand (2.2), Romania (1.9), and Kazakhstan (2.0). However, China (20,871), the United States (9123) and Italy (4075) bore the brunt of the deaths, which altogether accounted for more than half of the global IDU-attributable liver cancer deaths.

### 3.4. The Results of Spatial Autocorrelation

The global Moran’s I index and its *Z* scores and *p*-values in mortality rates of IDU-attributable cirrhosis and IDU-attributable liver cancer from 1990 to 2015 at the country level are shown in Table 1. Moran’s I index for IDU-attributable liver cancer mortality were small, and ranged between 0.10 and 0.11 in 1990, 1995, and 2000, with the corresponding *Z* scores > 1.96, and *p*-values < 0.05, which indicated that mortality rates of IDU-attributable liver cancer were weakly spatial clustered. In the year of 2005, 2010, and 2016, the Global Moran’s I tests for mortality rate of liver cancer-IDU were not statistically significant (*Z* scores < 1.96, and *p*-values > 0.05), which indicated that mortality rates of IDU-attributable liver cancer trended to be random distributed among countries. By contrast, the results of the global Moran’s I tests for mortality rates of IDU-attributable cirrhosis were statistically significant for all the periods (*Z* scores ≥ 2.58, and *p*-values < 0.01), with Moran’s I index ranged between 0.46 and 0.59, which indicated a strong clustering tendency of IDU-attributable cirrhosis mortality.

**Figure 7:**
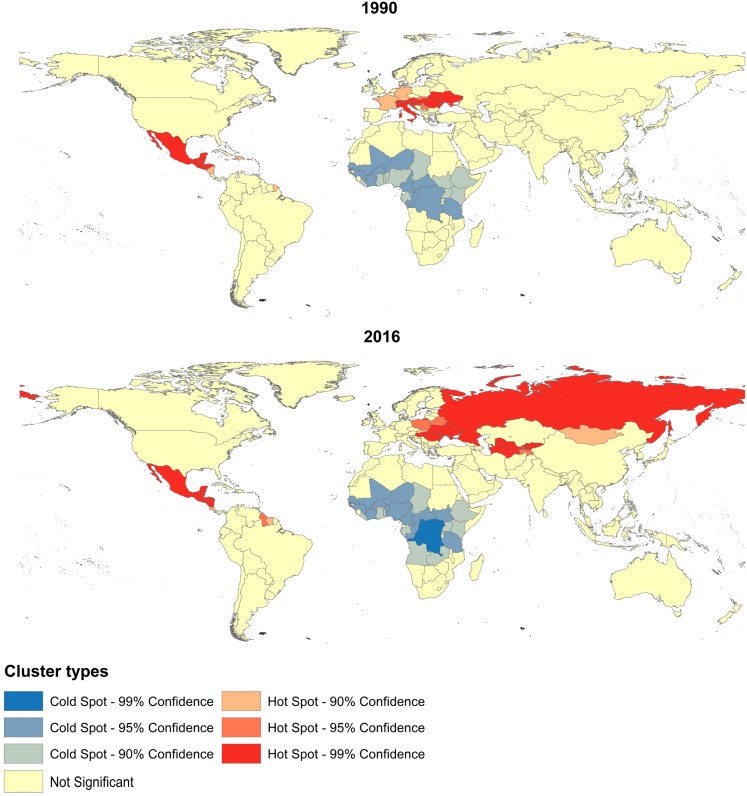
Local spatial autocorrelation for IDU-attributable cirrhosis mortality among 195 countries, 1990–2016.

In order to recognize the location of cluster of countries with high mortality rate of IDU-attributable cirrhosis, namely hot spots of IDU-attributable cirrhosis mortality, the Getis-Ord Gi* statistic/the local Gi*(d) statistic was done for IDU-attributable cirrhosis mortality of 195 country from 1990 to 2016 (Figure 7). In 1990, three clusters were identified, one cold spot (low mortality—low mortality clusters) and two hot spots (high mortality—high mortality clusters). Hot spots clustered in Latin America (Mexico, Guyana, Belize, Honduras, Nicaragua, EI Salvador, and Nicaragua) and Eastern Europe and Central Europe (Ukraine, Romania, Moldova, Hungary, Poland, and Belarus). Up to 2005, Central Asia (Uzbekistan, Kazakhstan, Turkmenistan, and Tajikistan) grew into a new hot spot, and Russia became new member of the Central Europe hot spot. Cold spots of IDU-attributable cirrhosis mortality stayed relatively stable from 1990 to 2016 and were mainly located in South Africa.

## 4. Discussion

Worldwide, IDU has become an increasing larger contributor to the mortality of cirrhosis and liver cancer. Our study provides a global overview of the mortality trends of IDU-attributable cirrhosis and IDU-attributable liver cancer from 1990 to 2015, and interprets the trends from the aspects of age effects, period effects and cohort effects. Meanwhile, our study probes into the spatial distribution patterns of IDU-attributable cirrhosis and liver cancer mortality, and shows the clustering distribution of most-at-risk areas. To our knowledge, our study is the first attempt to applied APC model analysis and spatial autocorrelation analysis to mortality pattern of IDU-attributable advanced liver disease.

The APC model analysis indicated a positive period effect and age effect, whereas an overall negative cohort effect on the mortality of IDU-attributable cirrhosis and IDU-attributable liver cancer. Furthermore, the increases of mortality from IDU-attributable cirrhosis and IDU-attributable liver cancer were mainly driven by period effects, since period effect increased continuously during the whole period and echoed the mortality trends.

Period effects reflected the instant effects of social factors on disease mortality. In our study, period effects increased by 6.82-fold for IDU-attributable cirrhosis and 3.08-fold for IDU-attributable liver cancer from 1990 to 2015. These increases, on the one hand, were a result of an accumulating exposure to HBV/HCV in the early stage, since cirrhosis and liver cancer death generally occurred 20–30 years after HBV/HCV infection [33,34]. In the late 1960s, the USA experienced a heroin epidemic, which extended to the late 1970s [35,36]. During the 1970s, drug abuse also grew rapidly in some European countries and South-East Asia countries, with an increasing trend towards injecting use of heroin [37,38]. In addition, the disposable syringes and needles came into general use around 1960 [39], which might have facilitated the epidemic of injecting drug use.

In sharp contrast to the boosted drug abuse, however, the global response to the increasing transmission risk of HBV/HCV among drug users was tardy. However the association of subcutaneous or intravenous IDU with homologous serum hepatitis (named as hepatitis B virus in 1976) was first reported as early as 1950 [40,41,42], yet the risk factors have not been well elucidated, and researches were conducted on a small scale. It was not until the isolation of HBV in 1976 and the isolation of HCV in 1989, that people really pay attention to the high risk of acquisition and transmission of blood-borne virus among IDUs [43,44,45,46,47]. Furthermore, the uptake of prevention and control measures against HBV/HCV transmission among IDUs lagged significantly behind people’s recognition of the transmission risk. Before 1970 in the USA, most drug addicts were sequestered in mental hospitals and jails, if they received any attention at all [48]. Thus, during the 1970s heroin epidemic, IDUs expose to uncontrolled HBV/HCV infection risk.

On the other hand, for those who have infected HBV/HCV, rate of treatment remained low. Efficacious treatments for HCV have been available for years, and more than 90% of hepatitis C can be cured with oral antivirals [49]. However, access to these antivirals is very low, with only 1–2% of HCV infected IDUs being access to treatment annually [50,51,52,53,54]. The situation is similar for chronic HBV infection, for which highly potent and well-tolerated oral antivirals have been available for more than a decade [55,56], whereas access to these drugs remains low in many countries [14]. Therefore, the explosive growth of IDU without interventions during the 1970s and limited coverage of treatment services among drug users were responsible for the substantial increase of the period effects.

The age effect reflected the change of mortality risk with age. In our study, age effects on IDU-attributable cirrhosis peaked in 45–49 years of age, with the mortality risk of 47.78-fold relative to 15–19 years of age; and age effects on IDU-attributable liver cancer peaked in 60–64 years of age, with the mortality risk of 36.63-fold relative to 15–19 years of age. These peaks of mortality risk seemed to fit with the natural history of liver disease since cirrhosis generally occurred 20–30 years after HBV/HCV infection [33,57], and liver cancer mostly occurred subsequent to cirrhosis [34]. Notably, however, the IDU-specific liver cancer mortality peaked in an earlier age in comparison with total liver cancer mortality. Specifically, total liver cancer mortality peaked in 75–79 years of age [58], whereas IDU-attributable liver cancer concentrated in 60–64 years of age. This “premature” death of liver cancer among IDUs was very likely due to a hepatotoxicity of the drugs. Evidence suggested that drug abuse can induce severe hepatotoxic effects and liver injury [59,60,61], which might have accelerated the progression of liver cancer and attendant mortality. In addition, the initiation of drug use usually peaks in young adulthood [62,63,64], and the incidence of hepatitis C is often high in younger IDUs [65,66]. Therefore, IDUs was very likely to have an earlier accumulation of risks compared with people dying of other risks-specific liver cancer, which partly explained the ‘’premature’’ death of IDU-specific liver cancer.

Cohort effects reflected variations in disease risk that applies to a certain birth cohort compared to surrounding birth cohorts, since people born in different years experienced different social or historical events during their life process and thus being exposed to different risks. An overall negative cohort effect was observed in both diseases, except two increases of mortality risk observed around the 1910–1934 and 1945–1959 birth cohorts, which matched the time of the First World War (1914–1918) and the Second World War (1939–1945). A shortage of medical care and a breakdown of accompanying socioeconomic progress undoubtedly contributed to the rise of mortality risk in people born during the war [67,68]. The mortality risk simply declined since after 1960, which to a large extent attributed to scientific progress and medical efforts on controlling drug abuse and HBV/HCV infection, including the Needle and syringe programs (NSP), the opiate substitution treatment (OST), the mass hepatitis B vaccination programs, and screening programs of HCV [6,69,70]. Besides, the information, education and counseling (IEC), the provision of injection paraphernalia, and supervised injecting facilities (SIFs) have been carried out successively in recent years, which are partly effective in reducing HCV transmission and injection risk behavior (IRB) [71].

Both HIV and HBV/HCV are international spread of blood-borne viruses by IDU. IDU-related HIV and AIDS are noted for their geographical clustering [72,73], while our results suggested that, IDU-related cirrhosis was also geographically clustered. By comparison, IDU-related liver cancer trended to be random distributed, since IDU made a small contribution to liver cancer burden in most of the countries, and only Mongolia, Zimbabwe, Italy, Egypt, France, Thailand, Romania, and Kazakhstan had a mortality rate higher than 2/100,000 population.

The cluster regions of IDU-attributable cirrhosis mortality had two characteristics. On the one hand, as expected, hot spots of IDU-attributable cirrhosis mortality were highly overlapped with drug-consumption regions, drug-producing regions and drug trafficking routes. Central Europe especially Russia and Ukraine, were high drug consumption markets with 1.27 percent of the population aged 15–64 injecting drugs, which accounted for almost one in four (24 percent) of the total number of IDUs worldwide; Central Asia was adjacent to the Golden Crescent and was the main conduit for the Afghan drug trafficking out, and the prevalence of injecting drug use was also high (0.72 percent of the population aged 15–64); Latin America was one of the world’s largest illicit drug producers, and Mexico was the major drug distributing center for Latin American drugs to the United States and its own consumer market [10,74]. Since drug abuse has been closely linked to HCV transmission, countries where drug use is highly prevalent as well as countries cultivate or trafficking drugs usually have higher drug consumption and ‘pockets’ of HBV/HCV infections may nevertheless occur considering the huge number of potential susceptible drug users.

On the other hand, as observed, hot spots of IDU-attributable cirrhosis mortality were mainly located in middle-to-low income countries. Injection drug use represents the core of the HCV epidemic in many settings particularly high-income countries [11,75], advanced liver disease mortality resulting from HCV infection via IDU, however, clustered in low- and middle-income countries. The main reason for this phenomenon is the significant gaps in prevention and treatment services among countries, which is largely dictated by economic conditions [71,76]. Indeed, HCV infection have stabilize in most of the high-income countries, and even declined among some resources rich European countries such as the United Kingdom, Germany, France and Italy [7,77], despite the facts that these countries have a long history of drug use and have a high level of drug consumption. The stabilization of HCV infection in those resources rich Europe countries to a large extent could attribute to the high coverage of primary interventions including Needle syringe programs (NSP) and opioid substitution therapy (OST) [78]. However, coverage of these interventions among IDUs in resource poor settings is negligible [54,76]. The situation is similar for the treatment of HCV. Efficacious treatments for HCV have been available for years, whereas access to these antivirals remains very low in many countries especially resource poor settings due to the high cost of HCV antivirals [79,80,81,82]. Therefore, IDU was the initiator of HCV transmission, whereas limited coverage of harm reduction services and antivirals was the chief culprits to blame for the development of advanced liver disease.

To clarify, as with HBV/HCV, hepatitis delta virus (HDV) infection is also known to be prevalent in IDUs. HDV is a small, defective RNA virus that can infect only individuals who have HBV [83]. Worldwide, 350 million people are HBV infected, of which 4% are co-infected with HDV [84]. Patients with HBV and HDV co-infection have more severe liver disease [85,86] and more rapid progression to advanced liver disease [87,88,89] than do those with HBV infection alone. Though prevalence of HDV is declining in some endemic areas, yet increasing in northern and central Europe because of immigration [90]. Therefore, HDV might be a contributor to the high IDU-attributable cirrhosis mortality in northern and central Europe such as Moldova and Romania.

## 5. Conclusions

IDU has become an increasingly larger contributor to the global cirrhosis and liver cancer mortality. Both the high risk of hepatitis C infection among drug users and the limited coverage of prevention and treatment services were responsible for the substantial increase in the mortality. Areas of particular concern are low- and middle-income countries of Latin America, East and Central Europe and Central Asia, where drugs are prevalent, produced and trafficked. Further efforts are needed to ensure access to primary harm reduction services and essential antivirals, especially in low-income settings.

## Figures and Tables

**Table 1:** Global spatial autocorrelation of IDU-attributable cirrhosis and IDU-attributable liver cancer mortality among 195 countries, 1990–2016.

Year	Liver Cancer Mortality-IDU			Cirrhosis Mortality-IDU		
	Moran’s I	*Z* Score	*p*	Moran’s I	*Z* Score	*p*
1990	0.10	2.21	0.027	0.55	8.96	<0.001
1995	0.11	2.40	0.016	0.60	9.79	<0.001
2000	0.10	2.30	0.021	0.66	10.66	<0.001
2005	0.07	1.93	0.054	0.66	10.59	<0.001
2010	0.06	1.80	0.072	0.63	10.20	<0.001
2016	0.06	1.93	0.054	0.64	10.26	<0.001

## Supplementary Materials

The following are available online at http://www.mdpi.com/1660-4601/15/1/170/s1, Table S1: Age-period-cohort model analysis results for global mortality of IDU-attributable cirrhosis, Table S2: Age-period-cohort model analysis results for global mortality of IDU-attributable liver cancer.
