# Supplementary material for: Global Mortality Burden of Cirrhosis and Liver Cancer Attributable to Injection Drug Use, 1990–2016: An Age-Period-Cohort and Spatial Autocorrelation Analysis
Source: Int J Environ Res Public Health. 2018 Jan 22;15(1):170. doi: 10.3390/ijerph15010170 (PMC5800269; doi:10.3390/ijerph15010170)
Supplement: Supplementary file 1 [file ijerph-15-00170-s001.pdf]

Table S1. Age-period-cohort (APC) model analysis results for global mortality of IDU-attributable cirrhosis.

Virables	Coef	95%CI	S.E.	RR	95%CI
Intercept	8.37	(8.36 – 8.38)	0.00		
Age					
15–19	–2.88	(–2.95, –2.82)	0.03	1.00	
20–24	–1.35	(–1.37, –1.32)	0.01	4.65	(4.49–4.82)
25–29	–0.51	(–0.53, –0.49)	0.01	10.70	(10.25–11.17)
30–34	0.15	(0.14, 0.17)	0.01	20.83	(19.87–21.83)
35–39	0.58	(0.57, 0.60)	0.01	32.03	(30.49–33.65)
40–44	0.86	(0.85, 0.87)	0.01	42.31	(40.20–44.53)
45–49	0.98	(0.97, 0.99)	0.01	47.78	(45.33–50.36)
50–54	0.98	(0.97, 0.99)	0.00	47.60	(45.12–50.22)
55–59	0.85	(0.84, 0.86)	0.00	41.84	(39.64–44.16)
60–64	0.65	(0.64, 0.66)	0.00	34.15	(32.35–36.04)
65–69	0.34	(0.33, 0.35)	0.01	25.21	(23.90–26.59)
70–74	–0.06	(–0.07, –0.05)	0.01	16.88	(16.03–17.78)
75–79	–0.59	(–0.61, –0.58)	0.01	9.88	(9.41–1.038)
Period					
1990	–0.59	(–0.59, –0.58)	0.00	1.00	
1995	–0.30	(–0.31, 0.29)	0.00	1.33	(1.33–1.33)
2000	–0.12	(–0.13, –0.12)	0.00	1.59	(1.58–1.59)
2005	0.14	(0.13, 0.14)	0.00	2.06	(2.06–2.07)
2010	0.33	(0.32, 0.33)	0.00	2.50	(2.49–2.50)
2015	0.54	(0.53, 0.55)	0.00	3.08	(3.08–3.09)
Cohort					
1915–1919	0.51	(0.47, 0.55)	0.02	1.00	
1920–1924	0.30	(0.27, 0.32)	0.01	0.81	(0.80–0.82)
1925–1929	0.43	(0.41, 0.45)	0.01	0.92	(0.90–0.94)
1930–1934	0.42	(0.40, 0.43)	0.01	0.91	(0.89–0.93)
1935–1939	0.29	(0.28, 0.31)	0.01	0.80	(0.78–0.82)
1940–1944	0.28	(0.26, 0.29)	0.01	0.79	(0.77–0.81)
1945–1949	0.10	(0.08, 0.11)	0.01	0.66	(0.64–0.68)
1950–1954	0.17	(0.15, 0.18)	0.01	0.71	(0.69–0.73)
1955–1959	0.23	(0.21, 0.24)	0.01	0.75	(0.73–0.77)
1960–1964	0.16	(0.15, 0.17)	0.01	0.70	(0.96–0.72)
1965–1969	0.04	(0.02, 0.05)	0.01	0.62	(0.61–0.64)
1970–1974	–0.14	(–0.16, –0.12)	0.01	0.52	(0.51–0.53)
1975–1979	–0.28	(–0.30, –0.26)	0.01	0.45	(0.44–0.46)
1980–1984	–0.37	(–0.39, –0.35)	0.01	0.41	(0.41–0.42)
1985–1989	–0.40	(–0.42, –0.37)	0.01	0.40	(0.40–0.41)
1990–1994	–0.45	(–0.48, –0.42)	0.02	0.38	(0.38–0.38)
1995–1999	–0.57	(–0.62, –0.52)	0.03	0.34	(0.34–0.34)
2000–2004	–0.71	(–0.86, 0.57)	0.07	0.29	(0.26–0.33)
Deviance	385.85				
AIC	16.05				
BIC	194.15				

Note: * $p < 0.05$; ** $p < 0.01$; *** $p < 0.001$; Coef.: Coefficient; S.E.: Standard error; RR: Relative risk; CI: Confidence interval; AIC: Akaike Information Criterions; BIC: Bayesian Information Criterions.

Table S2. Age-period-cohort (APC) model analysis results for global mortality of IDU-attributable liver cancer.

Virables	Coef	95%CI	S.E.	RR	95%CI
Intercept	6.83	(6.80 – 6.85)	0.01		
Age					
15–19	–2.48 ***	(–2.61, –2.34)	0.07	1.00	
20–24	–1.86 ***	(–1.95, –1.78)	0.04	1.85	(1.76–1.95)
25–29	–1.27 ***	(–1.33, –1.21)	0.03	3.33	(3.09–3.59)
30–34	–0.67 ***	(–0.72, –0.63)	0.02	6.07	(5.54–6.64)
35–39	–0.22 ***	(–0.26, –0.18)	0.02	9.57	(8.66–10.56)
40–44	0.24 ***	(0.21, 0.27)	0.02	15.07	(13.55–16.76)
45–49	0.65 ***	(0.62, 0.67)	0.01	22.78	(20.37–25.48)
50–54	0.95 ***	(0.93, 0.97)	0.01	30.66	(27.30–34.44)
55–59	1.09 ***	(1.08, 1.11)	0.01	35.53	(31.54–40.03)
60–64	1.12 ***	(1.11, 1.14)	0.01	36.63	(32.48–41.30)
65–69	1.01 ***	(0.99, 1.02)	0.01	32.54	(28.89–36.65)
70–74	0.85 ***	(0.83, 0.87)	0.01	27.93	(24.87–31.37)
75–79	0.60 ***	(0.57, 0.62)	0.01	21.60	(19.32–24.16)
Period					
1990	–0.72 ***	(–0.74, –0.70)	0.01	1.00	
1995	–0.41 ***	(–0.43, –0.40)	0.01	1.36	(1.36–1.37)
2000	–0.15 ***	(–0.16, –0.14)	0.01	1.78	(1.76–1.79)
2005	0.12 ***	(0.11, 0.13)	0.01	2.33	(2.31–2.35)
2010	0.41 ***	(0.40, 0.43)	0.01	3.11	(3.09–3.13)
2015	0.75 ***	(0.73, 0.76)	0.01	4.34	(4.32–4.36)
Cohort					
1915–1919	0.50	(0.45, 0.56)	0.03	1.00	
1920–1924	0.40	(0.36, 0.44)	0.02	0.90	(0.89–0.92)
1925–1929	0.58 ***	(0.55, 0.62)	0.02	1.08	(1.06–1.11)
1930–1934	0.61 ***	(0.58, 0.64)	0.01	1.11	(1.08–1.14)
1935–1939	0.55 ***	(0.52, 0.57)	0.01	1.05	(1.01–1.08)
1940–1944	0.51 ***	(0.49, 0.54)	0.01	1.01	(0.98–1.04)
1945–1949	0.41 ***	(0.38, 0.43)	0.01	0.91	(0.88–0.93)
1950–1954	0.50 ***	(0.47, 0.53)	0.01	0.99	(0.97–1.02)
1955–1959	0.59 ***	(0.56, 0.62)	0.02	1.09	(1.06–1.11)
1960–1964	0.44 ***	(0.41, 0.48)	0.02	0.94	(0.92–0.96)
1965–1969	0.30 ***	(0.26, 0.34)	0.02	0.82	(0.80–0.83)
1970–1974	0.12 ***	(0.07, 0.16)	0.02	0.68	(0.67–0.69)
1975–1979	–0.19 ***	(–0.25, –0.14)	0.03	0.50	(0.50–0.50)
1980–1984	–0.58 ***	(–0.64, –0.51)	0.03	0.34	(0.34–0.34)
1985–1989	–0.78 ***	(–0.86, –0.70)	0.04	0.28	(0.27–0.28)
1990–1994	–0.92 ***	(–1.02, –0.81)	0.05	0.24	(0.23–0.25)
1995–1999	–1.31 ***	(–1.48, –1.14)	0.08	0.16	(0.15–0.18)
2000–2004	–1.73 ***	(–2.10, –1.37)	0.19	0.11	(0.08–0.15)
Deviance	79.46				
AIC	10.70				
BIC	–112.23				

Note: * p < 0.05; ** p < 0.01; *** p < 0.001; Coef.: Coefficient; S.E.: Standard error; RR: Relative risk; CI: Confidence interval; AIC: Akaike Information Criterions; BIC: Bayesian Information Criterions.
